# Hotspot *KRAS* exon 2 mutations in CD166 positive colorectal cancer and colorectal adenoma cells

**DOI:** 10.18632/oncotarget.24921

**Published:** 2018-04-17

**Authors:** Hung Lai Wong, Lawrence Po Wah Ng, Su Pin Koh, Lawrence Wing Chi Chan, Evelyn Yin Kwan Wong, Vivian Weiwen Xue, Hin Fung Andy Tsang, Amanda Kit Ching Chan, Ka Yue Chiu, Wah Cheuk, Sze Chuen Cesar Wong

**Affiliations:** ^1^ Department of Health Technology and Informatics, Faculty of Health and Social Sciences, The Hong Kong Polytechnic University, Hong Kong Special Administrative Region; ^2^ Department of Pathology, Queen Elizabeth Hospital, Kowloon Central Cluster, Hospital Authority, Hong Kong Special Administrative Region

**Keywords:** colorectal cancer, colorectal adenoma, CD166, KRAS exon 2 mutations, immunohistochemical staining

## Abstract

Colorectal cancer (CRC) is the third most common cancer and the fourth leading cause of cancer deaths worldwide. Recent studies have shown that cancer stem cells (CSCs) are an important cause of tumor recurrence and metastasis. We hypothesized that CSCs marker CD166-positive CRC and colorectal adenoma (CAD) cells consist of more hotspot mutations than CD166-negative CRC and colorectal adenoma cells. To verify this, formalin fixed paraffin embedded tissue specimens from 42 patients each with CRC and CAD were recruited and CD166 immunohistochemical (IHC) staining followed by macrodissection was performed. DNA extracted was used for quantitative polymerase chain reaction detection on a somatic mutation array. Results showed that the immunoreactivity of CD166 protein had significant difference among CRC, CAD, and normal colorectal epithelial tissues (NCET) (*P* < 0.0001, Kruskal-Wallis test). Moreover, nucleotide changes were found in *APC*, *KRAS*, *P53*, *PIK3CA*, *FBXW7* and *SRC* genes. Among those genes, *KRAS* exon 2 mutations were validated in another cohort of 70 CRC and 72 CAD specimens. Results showed that the difference in percentage of *KRAS* exon 2 mutations between CD166 positive and CD166 negative CRC specimens was significant (*P* < 0.05, chi-square test). Long term follow-up of the CRC patients showed that CD166-positive KRAS exon 2 mutations was useful in discriminating CRC patients with worse outcome. This study has provided evidence that *KRAS* exon 2 mutations are concentrated in CD166-positive cancer cells, with prognostic significance in CRC, and those mutations are also detected in CAD.

## INTRODUCTION

Colorectal cancer (CRC) is the third most common cancer and the fourth leading cause of cancer deaths in the world [1]. The disease is highly curable if detected at an early stage. However, early CRC is mostly symptomless [2]. A variety of screening tests have therefore been investigated for early detection of CRC [3, 4]. Among them, faecal occult blood test has been the most extensively investigated, but it has low detection sensitivity on each round of screening [3, 4]. Colonoscopy and sigmoidoscopy are the gold standards for examination of the colon and rectum. However, the cost, the need of full bowel preparation and sedation, and the small but definite risk of perforation make them less suitable for a widespread population screening [3, 4]. Hence, there is a need to develop new non-invasive diagnostic methods for the detection and monitoring of CRC.

Carcinoembryonic antigen (CEA) is a widely used serum marker for CRC, but is unreliable in detecting postoperative recurrence [5]. Other biomarkers utilizing thymidylate synthase [6], vascular endothelial growth factor [7], loss of heterozygosity at 18q [8] and microsatellite instability [9] may either be prognostic or predictive of treatment response. However, they could not provide additional clinical values to the classical method using histopathologic features by pathologists. Imaging modalities such as positron emission tomography scan and magnetic resonance colonoscopy are useful in the prognosis of long-term survival of CRC patients, but the methods are too expensive for routine postoperative surveillance.

CRC is a heterogeneous disease. Twenty-five percent and 30% of CRC patients, present respectively with tumour-node-metastasis (TNM) stages II and III [10], have a high risk of post-operative recurrence [11]. Historically, their 3-year disease-free survival was about 45-55%, and the 5-year overall survival was only 60% among patients who were treated with surgery alone [12]. The use of adjuvant 5-fluorouracil (5-FU)-based chemotherapy improves the disease-free survival by an absolute margin of around 16-18%, and overall survival of around 10–12% [13]. Oxaliplatin further adds to the benefit of 5-FU [14]. However, both 5-FU and oxaliplatin have acute and long-term side effects, and not all CRC patients benefit from such treatment [14]. Therefore, these adjuvant chemotherapies should be applied carefully. TNM classification is the most commonly used method in making the therapeutic decision, but it is not reliable in identifying patients with “high-risk stage IIB” CRC who may need more aggressive adjuvant chemotherapy [6].

In the majority of cases, CRC develops over a long period of time through the adenoma-carcinoma sequence [15]. Therefore, successful detection and removal of pre-malignant colorectal adenoma (CAD) lesions can effectively prevent progression to CRC. Adenomas are categorized as either conventional adenomas or sessile serrated polyps [16]. Moreover, adenomas may be flat, sessile, subpedunculated with a very short stalk or pedunculated [16].

Cancer stem cells (CSCs) are a small subpopulation of tumor cells that are capable of initiating and maintaining tumor growth, as well as having the ability of self-renewal [17, 18]. CSCs have been found in various kinds of tumors, including CRC [19]. It is widely considered that the existence of drug-resistant CSCs and/or residual CSCs after surgery is an important cause of tumor recurrence and metastasis [20]. Hence, researchers have actively investigated the relationship between the number of CSCs within a tumor and patient prognosis.

One strategy to identify CSCs in colorectal tissues is to detect the surface markers on colorectal CSCs, such as CD133, CD44 and CD166 [20]. CD166, also known as activated leukocyte cell adhesion molecule (ALCAM), functions as a regulator of intercellular adhesion [21]. Hence, CD166 may play a role in facilitating invasion and adhesion of CSCs to nearby tissues. An elevated level of CD166 in CRC cells has been reported to be associated with shorten overall survival [21, 22]. However, the prognostic significance of CD166 level in CRC patients remains controversial because CD166 is also detected in normal colorectal mucosa, inflammatory and stromal cells [23], making the marker not specific enough for prognosis prediction.

One alternative approach of prognosis evaluation is to make use of CRC-associated DNA mutations. For example, the gain-of-function mutation in the oncogene *Kirsten rat sarcoma viral oncogene homolog* (*KRAS*) is one of the key mutations involving CRC progression and metastasis [24], and has been reported to be associated with poor patient survival [25]. Recently, *KRAS* mutations have also been suggested to be an adverse prognostic marker of metastasis [26]. CRC carried *KRAS* mutations metastasized to the liver more rapidly than tumors carried wild-type *KRAS* [26]. Liver metastases that contained *KRAS* mutations were also associated with higher mortality when compared to their wild-type counterpart [26]. Hence, DNA mutations are a potentially useful biomarker for the management of post-treatment patients.

Based on these findings, we hypothesize that by detecting tumor-associated mutations that present in CD166-positive cancer cells (CD166-pcc), a more accurate prediction of tumor recurrence and metastasis may be achieved. We reason that such potentially enhanced accuracy may due to a combined advantage that 1) CD166-pcc may be more relevant to recurrence and metastasis than those found in other parts of the tumor, and 2) tumor-specific mutations could act as a discriminating marker such that only tumor-derived cells, but not normal cells, could be specifically detected. Hence, we first identified CD166-pcc in CRC and CD166-positive adenoma cells (CD166-pac) in CAD followed by detecting their mutations after macrodissection of those cells. Among those mutations found, we selected *KRAS* mutation and detected the hotspot exon 2 mutations of *KRAS* genes in another cohort of CD166 stained CRC and CAD specimens using Sanger sequencing. Our objective was to examine the clinical significance of hotspot exon 2 *KRAS* mutations in CD166-pcc and CD166-pac.

The results generated from this study will lay down a solid foundation in using CD166-pcc and CD166-pac associated mutations in screening, diagnosis and prognosis of CRC patients.

## RESULTS

### Immunohistochemical staining

**Figure 1:**
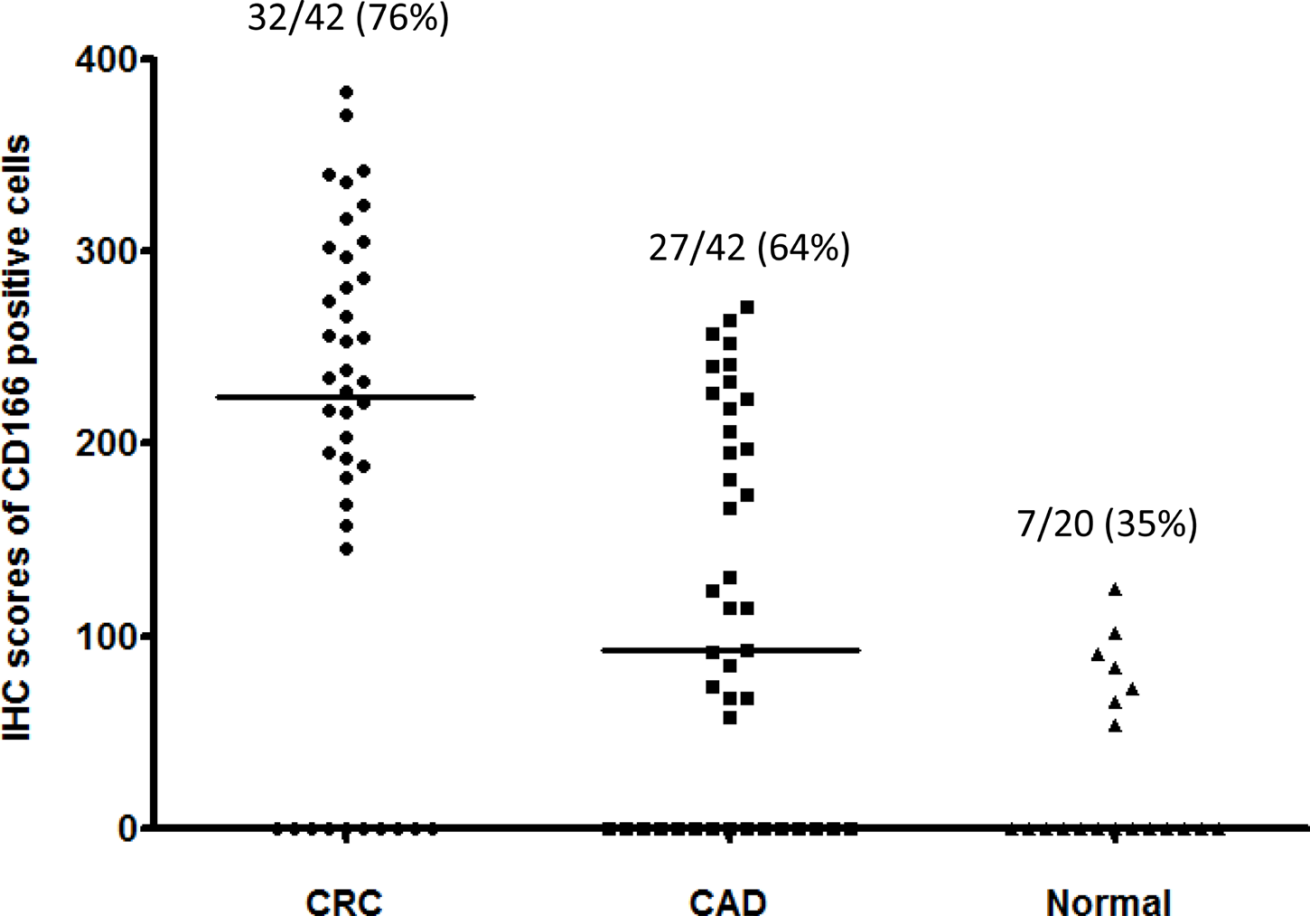
IHC scores of CD166-positive cells in 1) CRC, 2) CAD and 3) normal Normal: adjacent normal colorectal epithelial tissues; the median IHC scores of each group is indicated by a black horizontal line.

**Figure 2:**
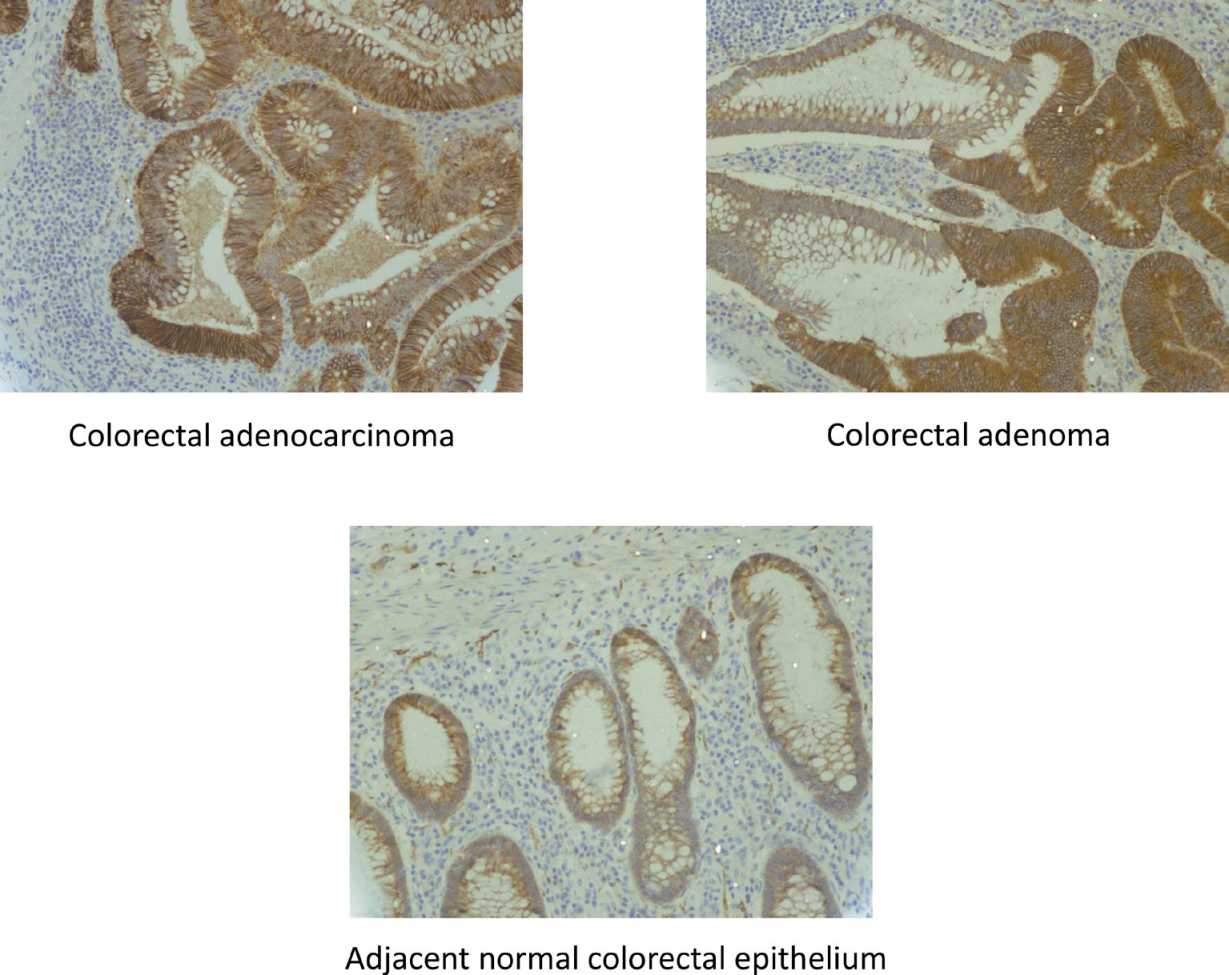
CD166 immunostaining in 1) CRC, 2) CAD and 3) adjacent normal colorectal epithelial tissues

CD166 protein was expressed in 76% (32/42) of CRC specimens, 64% (27/42) of CAD specimens and 35% (7/20) of normal colorectal epithelial tissues adjacent to CRC tissues (Figure 1). At the cellular level, CD166 protein was expressed in the membrane of the cell and representative photomicrograph of each specimen type in the first cohort was shown in Figure 2. Positive control of breast carcinoma showed intense positive cytoplasmic staining whereas negative control did not have any ICC staining. Detailed analysis showed that the immunoreactivity of CD166 protein, as shown by the immunohistochemical (IHC) scores, had significant difference among CRC, CAD, and normal colorectal epithelial tissues (Figure 1, *P* < 0.0001, Kruskal-Wallis test).

### Colon cancer mutation array test

Among the IHC stained slides, 12 CD166 positive CRC specimens, 6 CD166 negative CRC specimens, 30 CD166 positive CAD specimens and 8 CD166 negative CAD specimens were used for the colon cancer mutation array test. The criteria of selection were based on the quality and quantity of the DNA extracted.

In CRC, the number of CD166 positive specimens with nucleotide change is 6 (6/12 = 50.0%) whereas the number of CD166 negative specimens with nucleotide change is 2 (2/6 = 33.3%). In CAD, the number of CD166 positive specimens with nucleotide change is 24 (24/30 = 80%). Detailed analysis showed that 16 CD166 positive CAD specimens with 1 nucleotide change (16/30 = 53.3%) and 8 CD166 positive CAD specimens with 2 nucleotide change (8/30 = 26.7%) whereas the number of CD166 negative CAD specimens with nucleotide change is 4 (4/8 = 50%). Overall, there are a total of 44 nucleotide changes because 8 of the CD166 positive CAD specimens have 2 nucleotide change (Table 1).

**Table 1:** Nucleotide change in CD166 positive and CD166 negative CRC and CAD generated from colon cancer mutation array test

CRC	Mutants detected	One nucleotide change	Two nucleotide change	Total nucleotide change
CD166 positive specimens	FBXW7, TP53, KRAS	6/12 = 50.0%	0%	6
CD166 negative specimens	TP53	2/6 = 33.3%	0%	2
CAD				
CD166 positive specimens	APC, KRAS, SRC, TP53, PIK3CA	16/30 = 53.3%	8/30 = 26.7%	32
CD166 negative specimens	APC	4/8 = 50.0%	0%	4
Total nucleotide change		28	16	44

Mutation array results showed that nucleotide changes were found in *APC, KRAS, P53, PIK3CA, FBXW7* and *SRC* genes (Table 2). Out of the 44 nucleotide changes, 36 of them were found in CAD and only 8 of them were found in CRC. As discussed in the introduction, we focused our investigation by detecting hotspot mutations of *KRAS* exon 2 gene in another cohort of 70 CRC and 72 CAD specimens. After IHC staining, 49 of 70 (70%) CRC specimens were CD166 positive and 21 of 70 (30%) CRC specimens were CD166 negative. Similarly, 49 of 72 (68%) CAD specimens were CD166 positive and 23 of 72 (32%) CAD specimens were CD166 negative.

**Table 2:** Nucleotide and amino acid changes generated from the colon cancer mutation array test

Types	Gene	Nucleotide change	Amino acid change	Genotype
CD166 positive CRC				
Carcinoma	FBXW7	c.1393C>T	p.R465C	Mutant
Carcinoma	TP53	c.527G>T	p.C176F	Mutant
Carcinoma	KRAS	c.436G>A	p.A146T	Mutant
CD166 negative CRC				
Carcinoma	TP53	c.527G>A	p.C176Y	Mutant
CD166 positive CAD				
Adenoma	APC	c.4081_4082delCC	p.P1361fs*13	Mutant
Adenoma	APC	c.4391_4394delAGAG	p.E1464fs*8	Mutant
Adenoma	APC	c.4118_4118delC	p.P1373fs*42	Mutant
Adenoma	APC	c.4216C>T	p.Q1406*	Mutant
Adenoma	APC	c.3916G>T	p.E1306*	Mutant
Adenoma	APC	c.3956delC	p.P1319fs*2	Mutant
Adenoma	KRAS	c.436G>A	p.A146T	Mutant
Adenoma	KRAS	c.35G>A	p.G12D	Mutant
Adenoma	KRAS	c.34G>A	p.G12S	Mutant
Adenoma	SRC	c.1591C>T	p.Q531*	Mutant
Adenoma	TP53	c.473G>A	p.R158H	Mutant
Adenoma	PIK3CA	c.1633G>A	p.E545K	Mutant
CD166 negative CAD				
Adenoma	APC	c.4081_4082delCC	p.P1361fs*13	Mutant

### Sanger sequencing for *KRAS* exon 2 mutations

**Figure 3:**
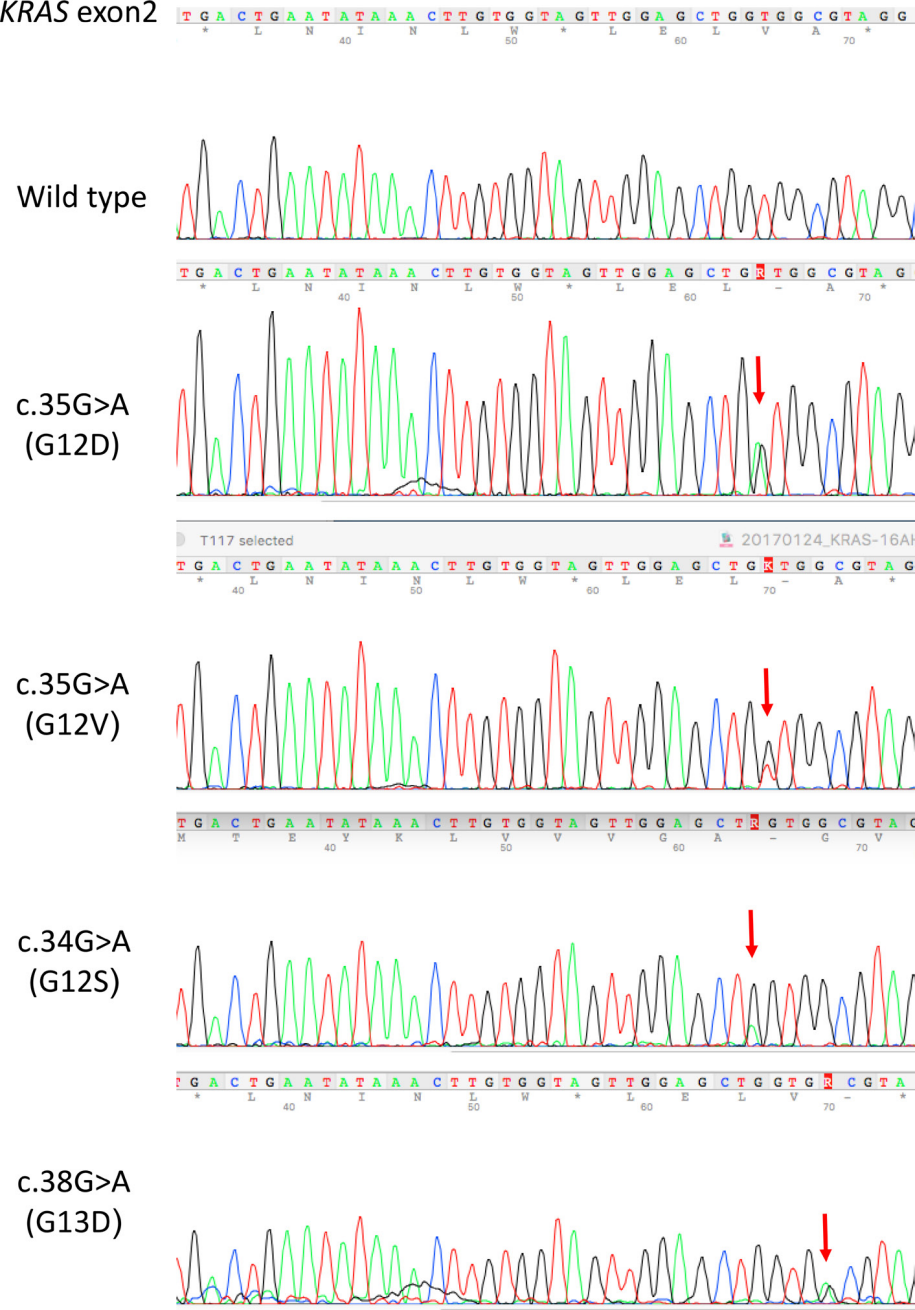
Representative *KRAS* exon 2 mutations in CRC and CAD FFPE tissues

*KRAS* exon 2 mutations were detected in 29 out of 49 CD166 positive (29/49 = 59%) CRC specimens and 7 out of 21 (7/21 = 33%) CD166 negative CRC specimens. On the other hand, *KRAS* exon 2 mutations were detected in 11 out of 49 CD166 positive (11/49 = 22%) CAD specimens and 4 out of 23 CD166 negative (4/23 = 17%) CAD specimens. The difference in percentage of *KRAS* exon 2 mutations between CD166 positive and CD166 negative CRC specimens is significant (*P* < 0.05, chi-square test) whereas whereas the difference of it between CD166 positive and CD166 negative CAD specimens is not significant (*P* = 0.62, chi-square test). Neglecting the status of CD166, the overall percentage of *KRAS* exon 2 mutations is 51% (36/70) in CRC and 21% (15/72) in CAD. Detailed analysis showed that the *KRAS* exon 2 mutations in CRC were composed of i) c.35G>A (G12D), ii) c.35G>T (G12V) and iii) c.34G>A (G12S). Moreover, c.35G>A (G12D) was detected in 18/49 (37%) CD166 positive CRC and 5/21 (28%) CD166 negative CRC; c.35G>T (G12V) was detected in 8/49 (16%) CD166 positive CRC and 2/21 (10%) CD166 negative CRC; c.34G>A (G12S) was detected in 3/49 (6%) CD166 positive CRC (Table 3). In CAD, *KRAS* exon 2 mutations were composed of i) c.35G>A (G12D), ii) c.35G>T (G12V), iii) c.38G>A (G13D) and iv) c.34G>A (G12S). Similarly, c.35G>A (G12D) was detected in 6/49 (12%) CD166 positive CAD and 1/23 (4%) CD166 negative CAD; c.35G>T (G12V) was detected in 5/49 (10%) CD166 positive CAD; c.38G>A was detected in 2/23 (9%) CD166 negative CAD and c.34G>A (G12S) was detected in 1/23 (4%) CD166 negative CAD (Table 4). Representative *KRAS* exon 2 mutations were shown in Figure 3. The distribution of *KRAS* mutation genotypes in various clinical stages of CRC and CAD patients were shown in Table 5 and the clinicopathological data of the studied patients were shown in Table 6.

**Table 3:** KRAS exon 2 mutations in CD166 positive and CD166 negative CRC

CRC	c.35G>A (G12D)	c.35G>T (G12V)	c.34G>A (G12S)	Total
CD166 positive	18/49 (37%)	8/49 (16%)	3/49 (6%)	29
CD166 negative	5/21 (24%)	2/21 (10%)	0/21 (0.0%)	7
Total	23	10	3	36

**Table 4:** *KRAS* exon 2 mutations in CD166 positive and CD166 negative CAD

CAD	c.35G>A (G12D)	c.35G>T (G12V)	c.38G>A (G13D)	c.34G>A (G12S)	Total
CD166 positive	6/49 (12%)	5/49 (10%)	0/49 (0%)	0/35 (0%)	11
CD166 negative	1/23 (4%)	0/23 (0%)	2/23 (9%)	1/23 (4%)	4
Total	7	5	2	1	15

**Table 5:** Distribution of KRAS exon 2 mutation genotypes in various clinical stages of patients

Mutations	Clinical stage	Total number
**CD166 positive CRC**		
c.35G>A	TNM stage III	12
	TNM stage IV	6
c.35G>T	TNM stage III	5
	TNM stage II	3
c.34G>A	TNM stage II	3
**CD166 negative CRC**		
c.35G>A	TNM stage II	3
	TNM stage I	2
c.35G>T	TNM stage III	2
**CD166 positive CAD**		
c.35G>A	Severe dysplasia	3
	Moderate dysplasia	3
c.35G>T	Moderate dysplasia	5
**CD166 negative CAD**		
c.35G>A	Severe dysplasia	1
c.38G>A	Moderate dysplasia	2
c.34G>A	Mild dysplasia	1

**Table 6:** Clinicopathological data of studied patients

Colorectal carcinoma patients (*n* = 70)	Number (%)
Sex	
Male	43 (61%)
Female	27 (39%)
Age	
Range	23-89 years old
Median	53.5 years old
TNM classification	
Stage I	13 (19%)
Stage II	22 (31%)
Stage III	25 (36%)
Stage IV	10 (14%)
**Colorectal adenoma patients (*n* = 72)**	**Number (%)**
Sex	
Male	39 (54%)
Female	33 (46%)
Age	
Range	24-74 years old
Median	48.5 years old
Degree in dysplasia	
Mild	24 (33%)
Moderate	28 (39%)
Severe	20 (28%)

### Prognostic significance of CD166 IHC staining and *KRAS* exon 2 mutations for post-treatment outcome of CRC patients

We further investigated the association of CD166 IHC staining and *KRAS* exon 2 mutations with the post-treatment outcome of CRC patients by following-up the CRC patients for 60 months. Among the 70 studied CRC patients, 25 of them had poor post-treatment outcome (14 patients were metastasized, 6 patients were relapsed and 5 patients were dead). When considering CD166 IHC staining alone, the marker showed a sensitivity of positive staining for 22/25 (88%) of the poor-treatment outcome patients. As there were a total of 49 patients with positive staining, hence 27/45 (60%) of the good-treatment outcome patients with positive staining was detected. In line with this logistic, negative CD166 staining was found in 3/25 (12%) poor-treatment outcome patients and 18/45 (40%) good-treatment outcome patients. Similarly, *KRAS* exon 2 mutations without macrodissection were detected in 9/25 (36%) poor-treatment outcome patients and 16/45 (36%) good-treatment outcome patients whereas *KRAS* exon 2 mutations without macrodissection were not detected in 16/25 (64%) poor-treatment outcome patients and 29/45 (64%) good-treatment outcome patients. When detecting *KRAS* exon 2 mutations in CD166 positive cells, positive *KRAS* exon 2 mutations in CD166-positive cells were detected in 19/25 (76%) poor-treatment outcome and 10/45 (22%) good-treatment outcome patients whereas negative *KRAS* exon 2 mutations in CD166-positive cells were detected in 4/25 (16%) poor-treatment outcome and 16/45 (36%) good-treatment outcome patients (Table 7). These results can show that positive *KRAS* exon 2 mutations in CD166 positive cells can help to distinguish patients with poor- from good-treatment outcome and have a much better sensitivity when compared with individual positive *KRAS* exon 2 mutations without macrodissection. However, CD166 positive IHC staining has the highest sensitivity in the prediction of the treatment-outcome of the patients (Table 7).

**Table 7:** The distribution of CD166 staining and CD166 *KRAS* exon 2 mutation status with the post-treatment outcome of CRC patients

	Poor-treatment outcome (25)	Good-treatment outcome (45)
CD166-positive staining	22/25 (88%)	27/45 (60%)
CD166-negative staining	3/25 (12%)	18/45 (40%)
Positive *KRAS* exon 2 mutations without macrodissection	9/25 (36%)	16/45 (36%)
Negative *KRAS* exon 2 mutations without macrodissection	16/25 (64%)	29/45 (64%)
Positive *KRAS* exon 2 mutations in CD166-positive cells	19/25 (76%)	10/45 (22%)
Negative KRAS exon 2 mutations in CD166-positive cells	4/25 (16%)	16/45 (36%)

### Correlation of CD166 and *KRAS* exon 2 mutations status with the clinico-histopathological factors of the patients

Multivariate regression showed that CD166 and *KRAS* exon 2 mutation statuses do not have any correlation to the clinical histopathological factors of the patients which include age, sex, tumor stage, lymph node status, distant metastasis and overall survival.

## DISCUSSION

The aim of this study was to examine whether CD166-pcc would consist of more hotspot mutations when compared to those CD166-ncc. If so, those hotspot mutations within the CD166-pcc may be used for diagnosis, prognosis as well as treatment benefit to CRC patients. The reason why we used this strategy because there is approximately only 36–40% of patients with CRC have tumor-associated *KRAS* mutations [27]. On the other hand, there are more than 60% of patients with CRC who do not have such *KRAS* mutations. Therefore, we hypothesized that more tumor-associated *KRAS* mutations related to drug-resistant or tumor recurrence and metastasis may be detected in those CSCs. This is a proof-of-concept study as CD166 is one of the CSC markers only. Similar to a previous study, our results showed that CD166 was expressed not only on the surface of epithelial cells within the stem cell niche, but also in the CRC cells and CAD cells [23]. It is now clear that sequential mutations of *APC, p53, Smad4* and *KRAS* genes are exclusively associated with the formation of CRC stem cells from the normal intestinal stem cells. These cancer stem cells, with CD166 as one of the major stem cell markers, are regarded as the primary sources for initiating CRCs [28]. In this study, our results showed that there were nearly double the number (1.8-fold more) of *KRAS* exon 2 mutations in those CD166-pcc (59%: 29/49) when compared to CD166-ncc in CRC (33%: 7/21). Detailed analysis demonstrated that the mutations c.35G>A (G12D) (37% vs 24%), c.35G>T (G12V) (16% vs 10%) and c.34G>A (G12S) (6% vs 0%) have a much higher prevalence in CD166-pcc than CD166-ncc. In contrast, a slightly higher percentage of *KRAS* exon 2 mutations was detected in CD166-pac (22%: 11/49) than CD166-negative adenoma cells (CD166-nac) (17%: 4/23) in CAD. More detailed analysis showed a higher detection rate of c.35G>A (G12D) (12% vs 4%) in CD166-pac than CD166-nac in CAD. In addition, c.35G>T (G12V) was only found in CD166-pac (10%) whereas c.38G>A (G13D, 9%) and c.34G>A (G12S, 4%) were only found in CD166-nac.

The majority of the tumor-associated *KRAS* mutations in CRC patients occur at codons 12, 13, and 61 of the *KRAS* gene. The result of these mutations is constitutive activation of *KRAS* signaling pathways [29]. It is now confirmed that patients with tumors harboring mutations in *KRAS* are unlikely to benefit from anti-EGFR antibody therapy, either as monotherapy [27] or in combination with chemotherapy [30]. On the other hand, *KRAS* exon 2, codon 12 and 13 mutations have important diagnostic and prognostic values in CRC [31–33]. As CRC is a disease that has a long progression period from a pre-malignant CAD stage, successful detection of hotspot mutations in CAD may provide a great benefit to the patients if this pre-malignant adenoma stage can be detected as earlier as possible.

This study produces significant results in four aspects: 1) c.35 G>A (G12D), c.35 G>T (G12V) and c.34 G>A (G12S) were detected in TNM stage II to IV CD166-pcc CRC specimens, 2) c.35 G>A (G12D), c.35 G>T (G12V), c.38G>A (G13D) and c.34G>A (G12S) were also detected in CAD, 3) a mutation c.34G>A (G12S) was found in both CRC and CAD specimens from the same patient and 4) prognostic significance of CD166-pcc *KRAS* exon 2 mutations in predicting poor treatment outcome. The first aspect can show that c.35 G>A (G12D), c.35 G>T (G12V) and c.34 G>A (G12S) are present in early and late TNM stages of CRC specimens. As there are nearly double the percentage of *KRAS* exon 2 mutations in those CD166-pcc in CRC specimens, we suggest that CD166 immunohistochemical staining followed by *KRAS* exon 2 sequencing can be performed on those specimens without *KRAS* exon 2 mutations for patient benefit in targeted therapy. The second aspect can show that *KRAS* exon 2 mutations have high potential to be an early CRC bio-marker. Although there was no significant difference in *KRAS* exon 2 mutations between CD166-pac and CD166-nac in CAD, an overall percentage of 21% (15/72) *KRAS* exon 2 mutations are still encouraging for us to detect other *KRAS* mutations in CAD. As the mutation c.34G>A (G12S) was found in both CRC and CAD specimens from the same patient, the clinical significance of it will be examined thoroughly in order to explore its potential in screening, detection and monitoring of CRC. Our preliminary findings on the prognostic significance of CD166 IHC staining and *KRAS* exon 2 mutations provide evidence that *KRAS* exon 2 mutations in CD166 positive cells would be useful in discriminating CRC patients with poor post-treatment outcome such as metastasis, relapse and death. Our results are different from a recent study which has shown that *KRAS* mutation increased the risk of lymph node involvement by 8 times in CD166-negative patients [34]. However, we noticed that a main difference between these 2 studies is that we macrodissected the CD166-pcc and CD166-ncc for *KRAS* exon 2 mutations detection whereas that study detected *KRAS* mutations using a tissue microarray block to represent the whole specimen. Therefore, the immunoreactivity of CD166 in the specimens in that study may be under-estimated. Nevertheless, the limitations of this study are 1) small sample size especially in colon cancer array and 2) only CD166 was examined and 3) only *KRAS* exon 2 mutations were examined. Therefore, a larger cohort of study is now being prepared to validate these results so as to determine the diagnostic, prognostic and therapeutic significance of these mutations. Moreover, more CSC markers and more tumor-associated mutations in genes such as *APC*, *p53* and *BRAF* will be examined. On the other hand, we are also preparing to detect those *KRAS* exon 2 mutations in the plasma of CRC patients by digital PCR or targeted re-sequencing. To study the functional relationship between CD166 and *KRAS* exon 2 mutations, we propose to use RNA interference–mediated knockdown of CD166 to compare the status of *KRAS* exon 2 mutations in CD166-positive and negative cells and examine the invasive potential of the CRC cells. This study has successfully provided evidence that 1) CD166-pcc has more *KRAS* exon 2 mutations than CD166-ncc in CRC and hotspot *KRAS* exon 2 mutations were detected in CAD and 2) these *KRAS* exon 2 mutations in CD166 positive cells may have prognostic significance in patients with CRC. We hope that these results can be verified in a large scale study and translated into clinical applications in future for CRC detection, monitoring and chemotherapeutic treatment response.

## MATERIALS AND METHODS

### Patient specimens for IHC staining

The first cohort of formalin fixed paraffin embedded (FFPE) specimens from 42 CRC patients and 42 CAD patients were recruited. Besides, 20 normal colorectal epithelial tissues were also included for comparison. CD166-pcc and CD166-pac were selected for human colon cancer somatic mutation polymerase chain reaction (PCR) array.

The second cohort of FFPE specimens from 70 CRC patients and 72 CAD patients were recruited. CD166-pcc and CD166-pac were selected for *KRAS* exon 2 mutations using Sanger sequencing. The study was approved by the Clinical Research Ethics Committee of the Queen Elizabeth Hospital, Kowloon Central Cluster, Hospital Authority, Hong Kong Special Administrative Region.

### Antibodies

CD166 (ALCAM)-specific antibodies (clone: MOG/07, Novocastra, UK) was used.

### Formalin fixed paraffin embedded tissue sectioning, IHC staining and evaluation

Serial tissue sections (4 μm thick) were cut and antigen retrieval was performed using Bond Epitope Retrieval Solution 2 on the Bond-max automated immunostainer (Vision BioSystems, Mount Waverley, Australia) at 100° C for 25 minutes. Staining was performed according to a standard protocol in the immunostainer. Polymer detection system was selected to avoid the problem of nonspecific endogenous biotin staining. Breast carcinoma was used as a positive control which was mounted on every test slide and negative controls were performed by replacing the antibody with tris buffered saline.

The stained slides were completely evaluated under light microscope at ×400 magnification by 2 independent observers without knowledge of clinical outcomes and in the case of disagreement, consensus was reached after thorough discussion and slides examination using a multi-headed microscope. All slides were scored semi-quantitatively and expressed as an IHC score by multiplying the “percentage of positive cells” and the “staining intensity”. Staining intensity was scored as follows: 0 = negative; 1 = weak; 2 = moderate; 3 = strong; and 4 = very strong. The IHC score ranged from 0 to 400. The scoring of percentage and staining intensity was targeted to 1) CRC cells, 2) CAD cells and 3) adjacent normal epithelial cells. Membranous staining was the expected result.

### Macrodissection

Thirty sections of each CD166 positive FFPE specimens were cut with 5 µm thickness per section. Each section was mounted on a superfrost slide. Microtome was cleaned with xylene before sectioning of each specimen in order to avoid any tissue carryover. The unstained sections of each specimen were deparaffinized with xylene followed by absolute alcohol. Selected areas on each slide were circled by comparing with a reference IHC stained slide of the same tissue section. Circled areas on each slide were filled with buffer ATL (Qiagen, Hilden, Germany) followed by scrapping using a new scalpel for each tissue specimen. The scrapped tissues were then transferred into an RNase-free microcentrifuge tube and the final volume was made up to 180 µl using buffer ATL. DNA extraction was performed according to instructions of a QIAamp DNA FFPE tissue kit (Qiagen).

### DNA extraction

Genomic DNA was extracted using the QIAamp DNA FFPE Tissue Kit (Qiagen) according to the manufacturer’s instructions with modifications. Briefly, macrodissected sections were deparaffinzed with xylene, followed by overnight proteinase K digestion. The mixture was then being loaded into the extraction column. DNA was eluted in 20 µl of water, and quantified by the QuantiFluor^™^ dsDNA system (Promega, Madison, USA).

### Human colon cancer somatic mutation PCR array

This array is a translational research tool that allows rapid, accurate, and comprehensive profiling of the top somatic mutations in human colon cancer specimens for the following genes: *APC, BRAF, CTNNB1/beta-catenin, FBXW7, KRAS, PIK3CA, SRC, and P53* (Cat: SMH-021AA, Qiagen, Germany). These mutations warrant extensive investigation to enhance the understanding of carcinogenesis and identify potential drug targets. This array includes 86 DNA sequence mutation assays designed to detect the most frequent, functionally verified, and biologically significant mutations in human colon cancer. These mutations are chosen from curated, comprehensive somatic mutation databases and peer-reviewed scientific literature, and represent the most frequently recurring somatic mutations compiled from over 29,000 colon cancer samples. The array layout and the gene table (Qiagen) were shown in Supplementary Tables 1 and 2, respectively.

The procedures involved DNA extraction (QIAGEN QIAamp DNA Mini Kit or FFPE Tissue Kit is recommended), quantitative PCR detection on qBiomarker Somatic Mutation PCR Array, and data analysis using the qBiomarker Somatic Mutation Data Analysis Template.

### qBiomarker mutation array procedures

Four specimens were tested in one qBiomarker Somatic Mutation PCR Array plate in each run. The protocol of qBiomarker Somatic Mutation PCR Array was followed. In brief, 700 ng genomic DNA extracted by QIAamp DNA FFPE Kit (Qiagen) was added to the master mix. Ten microliters of the reaction mix were used per reaction. The plate was then tightly sealed and centrifuged at 2000 rpm for 2 minutes and was put on ice until use. Real-time PCR was set up and run according to the protocol in a LightCycler 480 system (Roche Diagnostics, Mannheim, Germany).

Threshold cycle of each sample reaction was calculated using the cycle software. Linear view of the amplification plots was used to define the baseline value while log view of the amplification plots was used to define the threshold value. The threshold value was placed above the background signal but within the lower half to one-third of the linear phase of the amplification plot. The resulting threshold cycle values for all wells were exported to a blank excel spreadsheet and were formatted according to the excel-based PCR array Data Analysis Template which was available at https://www.qiagen.com/hk/shop/genes-and-pathways/data-analysis-center-overview-page/. Free data analysis software for qBiomarker Somatic Mutation PCR Array available at the above website was utilised for further investigation. The mutation array consists of the following genes:

### APC: 34 assays

The most commonly detected APC inactivation mutations are mainly composed of truncation mutations (due to nonsense mutations and frameshift mutations) and point mutations between codons 1250 and 1578.

### BRAF: 1 assay

In colon cancer, the BRAF mutation that leads to increased kinase activity, p. V600E mutation, is the most important to test.

### Beta-catenin (CTNNB1): 5 assays

The most frequently detected CTNNB1 mutations result in abnormal signaling in the WNT signaling pathway. The mutated codons are mainly several serine/threonine residues targeted for phosphorylation by GSK-3 beta.

### FBXW7: 1 assay

The mutations queried by these assays lay in either the third or fourth repeat of the protein’s WD40 domain, typically involved in protein-protein interactions.

### KRAS: 17 assays

The mutation assays include the most frequently occurring mutations in *KRAS* codons 12, 13, and 61. Mutations at these positions result in reduced intrinsic GTPase activity and/or cause *KRAS* to become unresponsive to RasGAP.

### PIK3CA: 7 assays

The most frequently occurring PIK3CA mutations mainly belong to two classes: gain-of-function kinase domain activating mutations and helical domain mutations that mimic activation by growth factors.

### SRC: 1 assay

SRC is a proto-oncogene and a tyrosine-protein kinase that plays a role in the regulation of embryonic development and cell growth. Mutations in this gene could be involved in the malignant progression of colon cancer.

### TP53: 20 assays

The most frequently detected somatic mutations in TP53 are largely composed of DNA-binding domain mutations which disrupt either DNA binding or protein structure.

### Sanger sequencing

The sequence of primers against *KRAS* exon 2 was designed as in Table 8.

**Table 8:** The sequence of primers against *KRAS* exon 2

Primer	Sequence	Product Size
Forward	GCCTGCTGAAAATGACTGAA	114
Reverse	TTGGATCATATTCGTCCACAA	114

BigDye Direct Cycle Sequencing Kit {Applied Biosystems (ABI), Thermo Fisher Scientific, Foster City, CA 94404, USA} and ABI Veriti thermal cyclers were used for amplification and cycle sequencing of *KRAS* gene. Each PCR reaction was consisted of i) 2.5 μL BigDye Direct PCR Master Mix, ii) 1 μL of 0.8 μM M13-tailed forward and reverse primer mix, iii) 1.5 μL genomic DNA (<2 ng/μL), or 1 μL of 2.0 ng/μL genomic DNA and iv) 0.5 μL deionized water to add up the total volume to 5 μL. Amplification and cycle sequencing of *KRAS* were conducted under the same condition. The PCR condition was shown as follows: 95° C for 10 minutes; 35 cycles each of i) 96° C for 3 seconds, ii) 62° C for 15 seconds and iii) 68° C for 30 seconds; and finally hold at 72° C for 2 minutes. Each PCR product was then diluted with 5 μL of water.

### Cycle sequencing

Each sequencing reaction consisted of 5 μL diluted PCR product, 1 μL of BigDye Direct Sequencing Master Mix (ABI) and 0.5 μL of BigDye Direct M13 Forward or Reverse Primer. Condition of cycle sequencing was shown as follows: 37° C for 15 minutes; 80° C for 2 minutes; 96° C for 1 minute; then 25 cycles each of i) 96° C for 10 seconds, ii) 50° C for 5 seconds and iii) 60° C for 75 seconds.

### Purification and sequencing

BigDye XTerminator Purification Kit (ABI) was used to purify the sequencing products. Each sequencing product was mixed with 36 μL SAM Solution and 8 μL XTermination solution on a spin plate and vortexed on IKA MS3 digital vortexer at 2000 rpm for 20 minutes. It was then centrifuged in a swinging-bucket centrifuge at 1000 g for 2 minutes. Sequencing was performed on Applied Biosystems 3500 Genetic Analyzer.

### Statistical analysis

The difference in IHC scores of CD166 protein among CRC, CAD and adjacent normal colorectal epithelial cells was studied using non-parametric Kruskal-Wallis test. Moreover, the association between CD166 status and *KRAS* exon 2 mutations was studied using chi-square test. Finally, multivariate regression (Cox proportional hazards regression) was used to analyze whether CD166 status, *KRAS* exon 2 mutations were correlated with the clinico- histopathological factors of the patients (Statistical Package for the Social Sciences Version 12.0 software, SPSS Inc., Chicago, IL., USA). A *P* value < 0.05 is considered to be statistically significant in all analyses. All *P* values are two-tailed.

## SUPPLEMENTARY MATERIALS TABLES




