# Supplementary material for: Hotspot KRAS exon 2 mutations in CD166 positive colorectal cancer and colorectal adenoma cells
Source: Oncotarget. 2018 Apr 17;9(29):20426–38. doi: 10.18632/oncotarget.24921 (PMC5945530; doi:10.18632/oncotarget.24921)
Supplement: Supplementary file 1 [file oncotarget-09-20426-s001.pdf]

Hotspot *KRAS* exon 2 mutations in CD166 positive colorectal cancer and colorectal adenoma cells

SUPPLEMENTARY MATERIALS

Supplementary Table 1: Array layout of the human colon cancer somatic mutation PCR array

Array Layout											
APC	APC	APC	APC	APC	APC	APC	APC	APC	APC	APC	APC
18852	13125	41617	18760	19203	18950	18764	18775	13113	18817	18700	18702
APC	APC	APC	APC	APC	APC	APC	APC	APC	APC	APC	APC
13129	41623	13121	19033	41619	18862	18834	19087	19088	18948	18836	13127
APC	APC	APC	APC	APC	APC	APC	APC	APC	APC	BRAF	CTNNB1
18873	18838	41618	19054	18786	19695	18561	13123	41620	13862	476	5675
CTNNB1	CTNNB1	CTNNB1	CTNNB1	FBXW7	KRAS	KRAS	KRAS	KRAS	KRAS	KRAS	KRAS
5664	6128	5667	5673	22932	553	554	555	517	518	516	521
KRAS	KRAS	KRAS	KRAS	KRAS	KRAS	KRAS	KRAS	KRAS	KRAS	PIK3CA	PIK3CA
522	520	528	529	527	531	532	533	534	19404	760	763
PIK3CA	PIK3CA	PIK3CA	PIK3CA	PIK3CA	SRC	TP53	TP53	TP53	TP53	TP53	TP53
764	766	773	775	776	1369	10790	10690	10739	10648	10687	10645
TP53	TP53	TP53	TP53	TP53	TP53	TP53	TP53	TP53	TP53	TP53	TP53
10705	10654	10758	10812	6932	43606	10656	10662	10891	10659	10660	10863
TP53	TP53	APC	BRAF	CTNNB1	FBXW7	KRAS	PIK3CA	SRC	TP53	SMPC	SMPC
10704	10768	99000030	99000006	99000042	99000063	99000008	99000012	99000071	99000041	99000017	99000017

Supplementary Table 2: Gene table of the human colon cancer somatic mutation PCR array. See Supplementary_ Table_2
