# Supplementary material for: Hotspot KRAS exon 2 mutations in CD166 positive colorectal cancer and colorectal adenoma cells
Source: Oncotarget. 2018 Apr 17;9(29):20426–38. doi: 10.18632/oncotarget.24921 (PMC5945530; doi:10.18632/oncotarget.24921)
Supplement: Supplementary file 2 [file oncotarget-09-20426-s002.doc]

Supplementary Table 2: Gene table of the human colon cancer somatic mutation PCR array

| Gene Table |
| --- |
| | Position | Gene | COSMIC ID | Nucleotide Change | Amino Acid Change | Assay Catalog # | | --- | --- | --- | --- | --- | --- | | A01 | APC | 18852 | c.2626C>T | p.R876* | SMPH000580A | | A02 | APC | 13125 | c.3340C>T | p.R1114* | SMPH000586A | | A03 | APC | 41617 | c.3700_3700delA | p.S1234fs*31 | SMPH018165A | | A04 | APC | 18760 | c.3916G>T | p.E1306* | SMPH000731A | | A05 | APC | 19203 | c.3919_3920insA | p.I1307fs*8 | SMPH001187A | | A06 | APC | 18950 | c.3920_3924delTAAAA | p.I1307fs*6 | SMPH000728A | | A07 | APC | 18764 | c.3921_3925delAAAAG | p.E1309fs*4 | SMPH000606A | | A08 | APC | 18775 | c.3925G>T | p.E1309* | SMPH000522A | | A09 | APC | 13113 | c.3927_3931delAAAGA | p.E1309fs*4 | SMPH000523A | | A10 | APC | 18817 | c.3934G>T | p.G1312* | SMPH000565A | | A11 | APC | 18700 | c.3956delC | p.P1319fs*2 | SMPH000576A | | A12 | APC | 18702 | c.3964G>T | p.E1322* | SMPH000685A | | B01 | APC | 13129 | c.4012C>T | p.Q1338* | SMPH000527A | | B02 | APC | 41623 | c.4081_4082delCC | p.P1361fs*13 | SMPH018172A | | B03 | APC | 13121 | c.4099C>T | p.Q1367* | SMPH000702A | | B04 | APC | 19033 | c.4110_4111delAA | p.P1372fs*2 | SMPH001245A | | B05 | APC | 41619 | c.4118_4118delC | p.P1373fs*42 | SMPH018167A | | B06 | APC | 18862 | c.4132C>T | p.Q1378* | SMPH000590A | | B07 | APC | 18834 | c.4135G>T | p.E1379* | SMPH000530A | | B08 | APC | 19087 | c.4216C>T | p.Q1406* | SMPH000690A | | B09 | APC | 19088 | c.4219_4220delAG | p.S1407fs*1 | SMPH001089A | | B10 | APC | 18948 | c.4233delT | p.S1411fs*4 | SMPH000571A | | B11 | APC | 18836 | c.4285C>T | p.Q1429* | SMPH000549A | | B12 | APC | 13127 | c.4348C>T | p.R1450* | SMPH000539A | | C01 | APC | 18873 | c.4385_4386delAG | p.S1465fs*3 | SMPH000704A | | C02 | APC | 18838 | c.4391_4394delAGAG | p.E1464fs*8 | SMPH000811A | | C03 | APC | 41618 | c.4463_4466delTATT | p.L1488fs*18 | SMPH018166A | | C04 | APC | 19054 | c.4473delT | p.F1491fs*16 | SMPH000577A | | C05 | APC | 18786 | c.4476delC | p.T1493fs*14 | SMPH000633A | | C06 | APC | 19695 | c.4660_4661insA | p.T1556fs*3 | SMPH001074A | | C07 | APC | 18561 | c.4666_4667insA | p.T1556fs*3 | SMPH000579A | | C08 | APC | 13123 | c.4729G>T | p.E1577* | SMPH001200A | | C09 | APC | 41620 | c.4731_4734delATGT | p.C1578fs*71 | SMPH018169A | | C10 | APC | 13862 | c.904C>T | p.R302* | SMPH000669A | | C11 | BRAF | 476 | c.1799T>A | p.V600E | SMPH001828A | | C12 | CTNNB1 | 5675 | c.109T>G | p.S37A | SMPH003985A | | D01 | CTNNB1 | 5664 | c.121A>G | p.T41A | SMPH003950A | | D02 | CTNNB1 | 6128 | c.133_135delTCT | p.S45del | SMPH004022A | | D03 | CTNNB1 | 5667 | c.134C>T | p.S45F | SMPH003953A | | D04 | CTNNB1 | 5673 | c.98C>A | p.S33Y | SMPH003959A | | D05 | FBXW7 | 22932 | c.1393C>T | p.R465C | SMPH005382A | | D06 | KRAS | 553 | c.182A>T | p.Q61L | SMPH007544A | | D07 | KRAS | 554 | c.183A>C | p.Q61H | SMPH007540A | | D08 | KRAS | 555 | c.183A>T | p.Q61H | SMPH007546A | | D09 | KRAS | 517 | c.34G>A | p.G12S | SMPH007533A | | D10 | KRAS | 518 | c.34G>C | p.G12R | SMPH007534A | | D11 | KRAS | 516 | c.34G>T | p.G12C | SMPH007535A | | D12 | KRAS | 521 | c.35G>A | p.G12D | SMPH007531A | | E01 | KRAS | 522 | c.35G>C | p.G12A | SMPH007536A | | E02 | KRAS | 520 | c.35G>T | p.G12V | SMPH007537A | | E03 | KRAS | 528 | c.37G>A | p.G13S | SMPH007543A | | E04 | KRAS | 529 | c.37G>C | p.G13R | SMPH007549A | | E05 | KRAS | 527 | c.37G>T | p.G13C | SMPH007541A | | E06 | KRAS | 531 | c.38_39GC>AT | p.G13D | SMPH007589A | | E07 | KRAS | 532 | c.38G>A | p.G13D | SMPH007538A | | E08 | KRAS | 533 | c.38G>C | p.G13A | SMPH007542A | | E09 | KRAS | 534 | c.38G>T | p.G13V | SMPH007545A | | E10 | KRAS | 19404 | c.436G>A | p.A146T | SMPH007562A | | E11 | PIK3CA | 760 | c.1624G>A | p.E542K | SMPH010629A | | E12 | PIK3CA | 763 | c.1633G>A | p.E545K | SMPH010627A | | F01 | PIK3CA | 764 | c.1634A>G | p.E545G | SMPH010633A | | F02 | PIK3CA | 766 | c.1636C>A | p.Q546K | SMPH010628A | | F03 | PIK3CA | 773 | c.3129G>T | p.M1043I | SMPH010695A | | F04 | PIK3CA | 775 | c.3140A>G | p.H1047R | SMPH010630A | | F05 | PIK3CA | 776 | c.3140A>T | p.H1047L | SMPH010632A | | F06 | SRC | 1369 | c.1591C>T | p.Q531* | SMPH014190A | | F07 | TP53 | 10790 | c.455C>T | p.P152L | SMPH014958A | | F08 | TP53 | 10690 | c.473G>A | p.R158H | SMPH014957A | | F09 | TP53 | 10739 | c.481G>A | p.A161T | SMPH015187A | | F10 | TP53 | 10648 | c.524G>A | p.R175H | SMPH014921A | | F11 | TP53 | 10687 | c.527G>A | p.C176Y | SMPH015119A | | F12 | TP53 | 10645 | c.527G>T | p.C176F | SMPH014960A | | G01 | TP53 | 10705 | c.586C>T | p.R196* | SMPH014949A | | G02 | TP53 | 10654 | c.637C>T | p.R213* | SMPH014928A | | G03 | TP53 | 10758 | c.659A>G | p.Y220C | SMPH014964A | | G04 | TP53 | 10812 | c.722C>T | p.S241F | SMPH014924A | | G05 | TP53 | 6932 | c.733G>A | p.G245S | SMPH014940A | | G06 | TP53 | 43606 | c.734G>A | p.G245D | SMPH034441A | | G07 | TP53 | 10656 | c.742C>T | p.R248W | SMPH014929A | | G08 | TP53 | 10662 | c.743G>A | p.R248Q | SMPH014902A | | G09 | TP53 | 10891 | c.814G>A | p.V272M | SMPH014987A | | G10 | TP53 | 10659 | c.817C>T | p.R273C | SMPH014907A | | G11 | TP53 | 10660 | c.818G>A | p.R273H | SMPH014913A | | G12 | TP53 | 10863 | c.833C>T | p.P278L | SMPH015086A | | H01 | TP53 | 10704 | c.844C>T | p.R282W | SMPH014941A | | H02 | TP53 | 10768 | c.535C>T | p.H179Y | SMPH015208A | | H03 | APC | 99000030 | copy number | copy number | SMPH017192A | | H04 | BRAF | 99000006 | copy number | copy number | SMPH017168A | | H05 | CTNNB1 | 99000042 | copy number | copy number | SMPH017204A | | H06 | FBXW7 | 99000063 | copy number | copy number | SMPH017225A | | H07 | KRAS | 99000008 | copy number | copy number | SMPH017170A | | H08 | PIK3CA | 99000012 | copy number | copy number | SMPH017174A | | H09 | SRC | 99000071 | copy number | copy number | SMPH038607A | | H10 | TP53 | 99000041 | copy number | copy number | SMPH017203A | | H11 | SMPC | 99000017 | positive PCR control | positive PCR control | SMPH017179A | | H12 | SMPC | 99000017 | positive PCR control | positive PCR control | SMPH017179A | |
|  |
